# Efficacy and Safety of Low-Dose Protamine in Reducing Bleeding Complications during TAVI: A Propensity-Matched Comparison

**DOI:** 10.3390/jcm12134243

**Published:** 2023-06-24

**Authors:** Kinan Kneizeh, Andrea Milzi, Felix Vogt, Klaus Witte, Nikolaus Marx, Michael Lehrke, Mohammad Almalla, Jörg Schröder

**Affiliations:** Department of Cardiology, Angiology and Critical Care (Medical Clinic 1), University Hospital RWTH Aachen, 52074 Aachen, Germany

**Keywords:** transcatheter aortic valve implantation (TAVI), protamine, vascular complications, bleeding

## Abstract

Objectives: We aimed to evaluate the efficacy and safety of low-dose protamine in reducing access site-related complications during Transcatheter Aortic Valve Implantation (TAVI) as compared to full-dose protamine. Background: Access site-related complications represent an independent predictor of poor outcomes of TAVI. Data regarding heparin reversal with protamine and the dosage needed to prevent bleeding complications are scarce among patients undergoing TAVI. Methods: A total of 897 patients were retrospectively included in the study. Patients who underwent percutaneous coronary intervention within 4 weeks before or concomitantly with TAVI (*n* = 191) were given 0.5 mg protamine for each 100 units of unfractionated heparin. All other patients (*n* = 706) were considered as a control group and 1 mg protamine for each 100 units of heparin was administered. Results: The combined intra-hospital endpoint of death, life-threatening major bleeding, and major vascular complications were significantly more frequent in patients receiving low-dose protamine [29 (15.2%) vs. 50 (7.1%), *p* < 0.001]. After propensity matching (*n* = 130 for each group) for relevant clinical characteristics including anti-platelet therapy [19 (14.6%) vs. 6 (4.6%), *p* = 0.006], low-dose protamine predicted the combined endpoint (OR 3.54, 95%-CI 1.36–9.17, *p* = 0.009), and even in multivariable analysis, low-dose protamine continued to be a predictor of the combined endpoint in the matched model (OR 3.07, 95%-CI 1.17–8.08, *p* = 0.023) alongside baseline hemoglobin. Conclusions: In this propensity-matched retrospective analysis, a low-dose protamine regime is associated with a higher rate of major adverse events compared to a full-dose protamine regime following transfemoral TAVI.

## 1. Introduction

The prevalence of aortic valve disease and particularly calcific aortic valve disease is increasing due to the aging population [1]. Since its introduction by Alain Cribier and colleagues in 2002 [2], transcatheter aortic valve implantation (TAVI) has become the primary therapeutic strategy for patients with symptomatic severe aortic stenosis who are at higher surgical risk or those older than 75 years [3], as well as for selected patients with severe aortic regurgitation [4].

Compared with surgical access such as the transapical approach, transfemoral access was associated with mortality and morbidity benefits that justify its use as a mainstream strategy [5,6]. However, despite the advancement of percutaneous closure device technology, access site complications (including bleeding and vascular complications) remain a major concern in transfemoral TAVI [7,8]. Vascular access site-related complications and bleeding are also predictors of poor procedural outcomes [7,9,10]. During the procedure, heparin administration is recommended to maintain an activated clotting time (ACT) of 300 s to reduce thromboembolic risk [11]. Heparin reversal, with protamine sulfate, is associated with reduced major and life-threatening bleeding events in the absence of an increase in thromboembolic events in patients undergoing TAVI [12]. However, the routine practice across centers is variable and data defining indications and the optimal dosing regimen of protamine after TAVI are still scarce.

Therefore, our retrospective analysis aimed to evaluate the efficacy and safety of low-dose protamine compared to full-dose protamine in preventing bleeding and vascular complications in patients undergoing transfemoral TAVI.

## 2. Methods

### 2.1. Study Population and Inclusion Criteria

**Figure 1:**
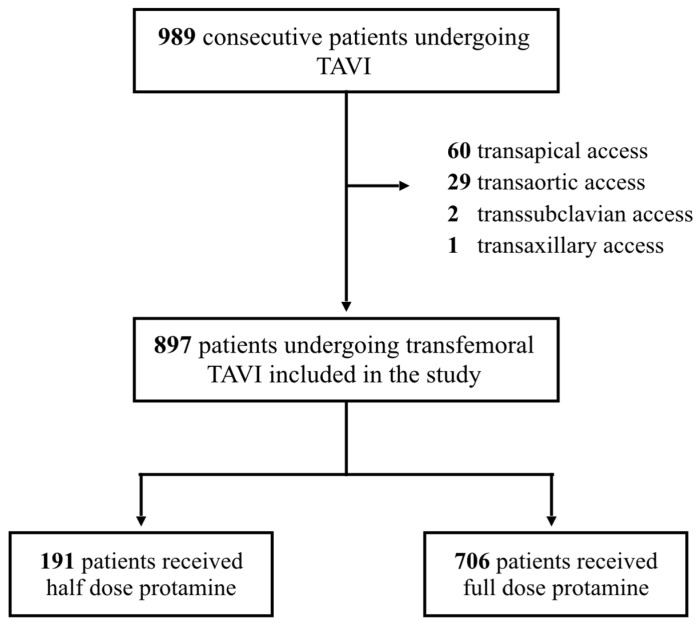
Study Population.

Between January 2015 and December 2020, patients admitted to the Department of Cardiology of the University Hospital of the RWTH Aachen due to severe aortic valve stenosis or severe aortic valve regurgitation who met the criteria for treatment based on the European Society of Cardiology guidelines on valvular heart disease [3] were discussed in our biweekly heart team meeting. The heart team includes an interventional cardiologist, an imaging cardiologist, a cardiovascular surgeon, and an anesthesiologist. Patients were evaluated based upon their medical records, including coronary angiography, pulmonary function, duplex Doppler sonography of the carotid arteries, transthoracic echocardiography, and transoesophageal echocardiography if needed. To estimate the cardiac operative risk, logistic EuroScore, STS-score, and Katz index were documented and patients were subsequently evaluated at the bedside by the heart team. Out of a total of 989 patients accepted for TAVI, 92 were excluded from the current analysis due to non-transfemoral approaches (transapical *n* = 60, transaortic *n* = 29, transsubclavian *n* = 2, transaxillary *n* = 1). All patients undergoing TAVI via the femoral artery (*n* = 897) were included in this retrospective study. Further details including patient disposition are demonstrated in Figure 1.

### 2.2. Ethical Approval

The protocol for this study as a retrospective analysis of routinely collected data was approved by the local ethics committee (EK 481/21). The study was performed in accordance with the ethical standards defined by the latest version of the Declaration of Helsinki.

### 2.3. Transcatheter Aortic Valve Implantation (TAVI)

The procedure was carried out by an interventional cardiologist, a cardiothoracic surgeon, and an anesthesiologist. TAVI procedures in our center are carried out under analgesic sedation. Femoral arterial access was guided by using a preoperative computed tomography angiogram of the pelvic vasculature. According to our standard operating procedure, weight-adapted unfractionated heparin was administered before placement of the TAVI introducer sheath. ACT was measured every 20 min and additional heparin was given to maintain ACT above 300. Closure of the access site was performed using either Prostar, Manta, or ProGlide devices. An additional pressure bandage was applied for 12 h following the intervention. Dual anti-platelet therapy was prescribed for 6 months.

### 2.4. Heparin Reversal

Heparin reversal took place after vascular closure in all patients. The standard dose was 1 mg protamine for every 100 units of unfractionated heparin that were given in the last 30–60 min. In long procedures, 0.75 of protamine was additionally administered per 100 IU of heparin that were given earlier than 60 min. ACT measurements were done both before and after protamine administration. Indications for low-dose protamine included planned PCI during the index procedure or in the 4 weeks prior to the TAVI. ‘Low dose’ protamine was half the usual calculated dose.

### 2.5. Post-Interventional Care

After the procedure, patients were monitored for 24 h in the intermediate care unit. Transthoracic echocardiography was performed immediately after TAVI and before discharge from our center. All patients underwent a duplex Doppler sonography of the access site of the femoral artery.

### 2.6. Study Outcomes and Definitions

The classifications of the vascular access site and access-related complications were based on the standardized definitions for important clinical endpoints in TAVI as proposed by the updated consensus document of the Valve Academic Research Consortium from 2012 (VARC-2 criteria) [13]. Primary outcomes were in-hospital (pre-discharge) life-threatening bleeding, major bleeding, major vascular complications, or death from any cause, taken individually or as a combined endpoint. Secondary outcomes consisted of minor bleeding, minor vascular complications, stroke, and myocardial infarction.

### 2.7. Statistical Analysis

Continuous variables are reported as mean ± standard deviation if normally distributed and median (interquartile ratio) if not normally distributed. Normal distribution was assessed by visual histogram inspection as well as with the Kolgomorov–Smirnov test. Dichotomic variables are reported as a proportion (percentage). The comparison of baseline continuous variables was performed with a *t*-test for normally distributed variables and with a Mann–Whitney test for non-normally distributed variables; distributions of binary variables were compared with a chi-squared test. In order to assess the association of two different protamine doses with interventional outcomes and complications, we performed a univariable logistic analysis; results are expressed as an odds ratio (OR) with a 95%-confidence interval. In order to adjust for possible confounders, we performed multivariable logistic analysis for the prediction of the combined endpoint, including in the model all variables with *p* < 0.10 in the univariable analysis (EuroScore, COPD, peripheral artery disease, glomerular filtration rate, hemoglobin levels, LDL levels, coronary artery disease, dual antiplatelet therapy) and then performing backward selection.

To exclude the effects of different baseline characteristics between the two study groups, we performed propensity matching based on sex, age (with a tolerance of ±5 years), and dual antiplatelet therapy. We then repeated our analyses in the matched population.

Analysis was performed with SPSS software. Statistical significance was awarded for *p* < 0.05.

## 3. Results

### 3.1. Baseline Characteristics

A total of 897 patients were included. The median age was 82 (IQR 78-85) years. Men were represented significantly more in the low-dose protamine group at 58.1% (*p* = 0.005). The average logistic Euroscore, as a predictor for cardiac operative risk and mortality, was significantly higher in patients receiving the low-dose protamine scheme. These patients also had a higher prevalence of coronary artery disease (CAD) (100% vs. 59.5%, *p* ≤ 0.001) and dual anti-platelet inhibition (40.5% vs. 6.7%, *p* ≤ 0.001), while other clinical characteristics were comparable between both groups.

Propensity matching was performed on 130 patients from the low-dose protamine group and 130 patients from the full-dose group. After matching, both groups presented similar characteristics regarding the above-mentioned differences, except for CAD which remained higher in the low-dose protamine group. Importantly, no in-between group difference was present regarding anti-coagulatory and anti-platelet therapy. The baseline characteristics of matched patients are presented in Table 1. Patients in both groups received comparable doses of unfractionated heparin (9302 ± 2976 vs. 9024 ± 2471, *p* = 0.88). In the majority of the patients, A. femoralis was used as an additional vascular access site with no significant difference between both groups (78.8% vs. 83.2%, *p* = 0.502). There was no difference between the groups in terms of closure device used. (*p* = 0.574). Further procedural characteristics are presented in Table 2.

### 3.2. Association of Protamine Dose Scheme with Clinical Outcomes

The combined primary intra-hospital endpoint of death, life-threatening major bleeding, and major vascular complications occurred significantly more often in patients receiving low-dose protamine compared to patients treated with full-dose protamine [29 (15.2%) vs. 50 (7.1%), *p* < 0.001]. In particular, the low-dose protamine group presented a higher incidence of life-threatening [9 (4.7%) vs. 14 (2%), *p* = 0.034] and major bleeding events [12 (6.3%) vs. 16 (2.3%), *p* = 0.005]. No significant difference between both groups was observed for stroke and [4 (2.1%) vs. 11 (1.6%), *p* = 0.608] and myocardial infarction [1 (0.5%) vs. 2 (0.3%), *p* = 0.610], respectively.

Additional outcomes for both groups are presented in Table 3. A larger blood transfusion volume was observed in patients with low-dose protamine treatment (1.1 ± 3.1 vs. 0.5 ± 1.6 units, *p* < 0.001).

Univariable logistic regression analysis was performed to assess the association between low-dose protamine treatment and adverse clinical outcomes. Low-dose protamine treatment significantly predicted the combined endpoint (OR 2.35, 95%-CI 1.44–3.82, *p* < 0.001) and life-threatening (OR 2.44, 95%-CI 1.04–5.74, *p* = 0.040) and major bleeding events (OR 2.89, 95%-CI 1.34–6.22, *p* = 0.007) in Table 4. In univariable logistic regression analysis, EuroScore (OR 1.37 per 10%-variation, 95%-CI 1.13–1.65, *p* = 0.001) and pre-procedural hemoglobin (OR 0.69 per 1 g/dL, 95%-CI 0.59–0.79, *p* < 0.001) were able to predict the combined endpoint; details of the univariable analysis are reported in Supplementary Table S1. In multivariable analysis, low-dose protamine treatment remained a significant predictor of adverse outcome (OR 2.07, 95%-CI 1.23–3.47, *p* = 0.006) alongside with EuroScore and pre-procedural hemoglobin, as reported in Supplementary Table S2.

Since the two study groups differed significantly regarding relevant clinical characteristics at baseline, we performed propensity matching of 130 patients from the low-dose protamine group and 130 patients from the full-dose group. Both groups presented with similar characteristics regarding the above-mentioned differences, except for CAD which remained higher in the low-dose protamine group although rates of anticoagulation and anti-platelet therapy were similar. The baseline characteristics of matched patients are presented in Table 1.

**Figure 2:**
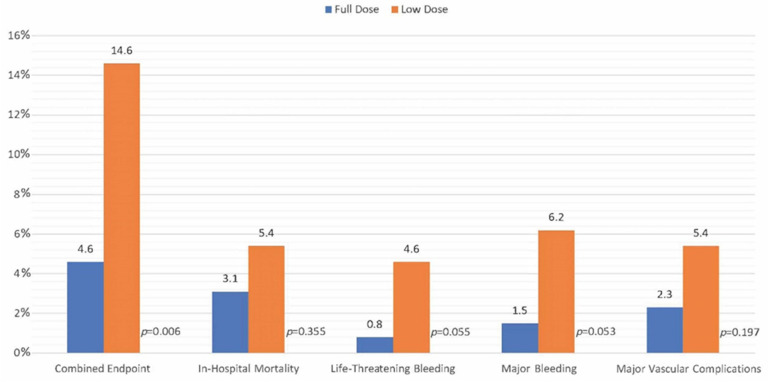
Primary and Secondary Outcomes in Matched Model.

Following propensity matching, low-dose protamine was still significantly associated with a higher incidence of the composite endpoint (OR 3.54, 95%-CI 1.36–9.17, *p* = 0.009). Details are reported in Figure 2, Table 5 and Table 6. In the univariable logistic regression analysis performed in the matched population, pre-procedural hemoglobin also reached statistical significance as a predictor of the combined endpoint (OR 0.71 per 1 g/dL, 95%-CI 0.56–0.89, *p* = 0.004); details of this analysis are shown in Supplementary Table S1. Even following propensity matching, in multivariable logistic regression analysis, low-dose protamine remained associated with a higher incidence of the composite endpoint (OR 3.07, 95%-CI 1.17–8.08, *p* = 0.023) together with pre-procedural hemoglobin (OR 0.73 per 1 g/dl, 95%-CI 0.58–0.93, *p* = 0.010). Details are shown in Supplementary Table S3.

## 4. Discussion

Transfemoral TAVI has emerged as an optimal therapy for high-risk patients with aortic stenosis and selected patients with aortic insufficiency. Access site-related complications remain the primary driver of post-interventional morbidity and mortality. In addition to closure devices, heparin reversal by protamine sulfate is an option to reduce access site bleeding, although a standardized dosing scheme during TAVI is lacking. Therefore, the aim of our study was to compare the efficacy of two regularly used doses of protamine in a retrospective analysis.

The major finding of our analysis is that full-dose protamine administration (1:1 protamine/heparin ratio) following transfemoral TAVI was associated with a lower incidence of the combined intra-hospital endpoint of all-cause mortality, major and life-threatening bleeding, as well as major vascular complications when compared with a low-dose protamine scheme (0.5:1 protamine/heparin ratio), mainly due to a significantly lower incidence of major and life-threatening bleeding. First, our data show an overall complication rate mostly comparable to major cohorts [14], especially when outcomes were defined according to VARC-2 criteria [7]. One of the largest registries of aortic stenosis treatment worldwide, the GARY registry, reported in-hospital mortality, major vascular complications, and major bleeding events in 5.2%, 4.1%, and 26.3% of patients following TAVI [15]. The rates of in-hospital mortality and major vascular complications were approximately equal to our data (4.1% for both). However, the rate of life-threatening and major bleeding in the GARY registry was considerably higher than that of our analysis (26.3% vs. 5.6%). It is likely that the difference in rates is accounted for by differences in definitions of major bleeding events. However, overall, our study cohort is representative of real-life data from larger registries.

Although the only existing single-center randomized clinical trial (PS TAVI) on protamine sulfate during TAVI, which was limited by its small sample size (*n* = 100), provided no evidence for a significant decrease in major and life-threatening bleeding complications among patients who routinely received protamine sulfate as compared to those in the placebo group [16], the use of protamine after cardiac and vascular interventions is routine practice [17,18]. In a meta-analysis of five trials including 6762 patients, protamine was associated with significantly less major bleeding after coronary angioplasty [19]. There also seems to be no greater rate of stent thrombosis after elective PCI when protamine is used [20]. A recent study reported protamine usage to lower significant bleeding and major vascular complications after TAVI without increasing the incidence of thromboembolic events [12]. More robust evidence on the efficacy of routine protamine administration versus selective protamine administration after TAVI is expected when the results of the ongoing ACE PROTAVI randomized double-blind trial are published. Notably, the optimal protamine dose required to prevent major bleeding events after TAVI has not been addressed. Evidence from the field of cardiac surgery suggests that a high dose of protamine sulfate (>1:1) can lead to adverse outcomes with respect to increased bleeding complications due to impaired hemostasis through the downregulation of thrombin generation as well as other side effects of protamine [21,22,23]. On the other hand, a lower dose of protamine (<0.6 mg per 100 IU of heparin) was associated with a reduced need for blood transfusion [20]. A protamine-heparin ratio between 0.6 to 1 was consequently suggested to provide optimal effects on hemostasis and bleeding during cardiothoracic operations [22], with a generally proposed dosing regimen of 1 IU per IU of heparin [24].

Our routine practice of the use of low-dose protamine in patients with recent PCI (0.5:1 protamine/heparin ratio) with the intention of balancing bleeding complications with stent thrombosis offered the opportunity to compare two doses of protamine in a real-world scenario. Patients receiving low-dose protamine experienced a significantly higher rate of the combined intra-hospital endpoint of death, life-threatening major bleeding, and major vascular complications compared with patients receiving full-dose protamine. This was driven by a significantly higher rate of both life-threatening and major bleeding events in the low-dose group, and translated into a higher blood transfusion volume as well as a longer hospital stay.

Whilst it is tempting to speculate that incomplete heparin reversal using the low-dose protamine scheme is insufficient in preventing access site bleeding in patients after TAVI, the conclusion should be tempered by the higher risk profile, as demonstrated by EuroScore and more frequent medication of anti-platelet and anticoagulant agents in those allocated low-dose protamine. In order to control for these baseline differences, we performed propensity matching between both groups, although this was only partially successful given that the primary indication for low-dose protamine was a recent or concurrent coronary intervention. Nevertheless, despite propensity matching, the primary combined endpoint as well as secondary endpoints such as minor bleeding, blood transfusion volume, and the length of hospital stay were still significantly greater in the low-dose group. A multivariable analysis determined that low-dose protamine alongside with EuroScore and baseline hemoglobin predicted the combined endpoint. Even after repeating the same analysis in the matched model, the combined endpoint was predicted only by low-dose protamine and baseline hemoglobin.

With respect to thrombosis, the overall rates of intervention-related stroke and myocardial infarction were extremely low in both study groups. We found no association between stroke rates and the dosage of protamine used. Stent thrombosis was confirmed only in one patient who received low-dose protamine after recent elective PCI, a further two cases of myocardial infarction due to coronary embolism were reported in patients in the full-dose protamine group. Overall, the extremely low rate of thromboembolic complications demonstrates that protamine application, independently of the suggested dosing scheme, seems not to be associated with thrombotic events.

## 5. Limitations

Despite being the first study assessing two different dosing schemes of protamine following TAVI, the main limitation is the retrospective nature of our analysis and the possible resultant selection bias which partially persists despite propensity matching for relevant clinical characteristics including anti-platelet therapy. Taking this into consideration highlights the role of conducting large randomized clinical trials to validate our results. Furthermore, in spite of the very low incidence of thromboembolic events following protamine administration in our cohort as well as in previous studies, the safety of a high-dose regime in patients who underwent a recent PCI still has to be assessed.

## 6. Conclusions

In this retrospective propensity-matched study of a large and unselected patient cohort, a low-dose protamine regime (0.5:1 protamine/heparin ratio) was associated with a higher rate of intra-hospital major adverse events compared to a full-dose protamine (1:1 protamine/heparin ratio) after transfemoral TAVI. This may suggest that a complete reversal of the heparin effect is needed to reduce bleeding complications after TAVI. These findings need to be confirmed in a prospective randomized trial.

## Figures and Tables

**Table 1:** Basic Characteristics of Patients Before and After Propensity Matching.

		Before Matching			After Matching		
	Overall (N = 897)	Protamine full dose (N = 706)	Protamine half dose (N = 191)	*p*-Value	Protamine full dose (N = 130)	Protamine half dose (N = 130)	*p*-Value
Median age (IQR)—year	82 (78–85)	81 (78–85)	82 (79–85)	0.481	80 (77–84)	82 (78–85)	0.065
Male—no. (%)	440 (49.1)	329 (46.6)	111 (58.1)	0.005	78 (60.0%)	78 (60.0%)	1.000
Median logistic EuroSCORE (IQR)—%	15 (10–23)	14.5 (10–22)	18 (11.4–26)	0.015	15.6 (9.6–23.0)	15.2 (10.5–25.5)	0.54
COPD—no. (%)	301 (33.3)	244 (34.4)	59 (30.1)	0.225	42 (32.3%)	44 (33.8%)	0.301
Diabetes mellitus—no. (%)	320 (35.7)	242 (36.1)	84 (44)	0.013	57 (43.8%)	44 (33.8%)	0.098
Hypertension—no. (%)	709 (79.1)	560 (79.3)	149 (78)	0.787	101 (77.7%)	98 (76.0%)	0.742
Peripheral artery disease—no. (%)	140 (15.6)	98 (13.8)	42 (22)	0.006	23 (17.7%)	35 (27.1%)	0.068
Cerebral artery disease—no. (%)	273 (30.5)	207 (29.2)	67 (35)	0.121	51 (39.2%)	45 (34.6%)	0.441
Smoker—no. (%)	101 (11.3)	75 (10.6)	26 (13.6)	0.246	18 (13.8%)	16 (12.3%)	0.713
Previous Stroke—no. (%)	100 (11.1)	79 (11.1)	21 (11)	0.590	18 (13.8%)	18 (13.8%)	1.000
Median GFR (IQR)—mL/min/1.73 m^2^	56.7 (41–73)	57 (41–74)	55.5 (39–69)	0.757	57 (41–71)	53 (38–69)	0.527
Median LDL (IQR)—mg/dL	84 (66–110)	86.5 (67–112)	80 (62–101.5)	0.901	85 (63–110)	78 (61–100)	0.135
Median Hemoglobin (IQR)—g/dL	11.9 (11–13)	11.9 (11–13)	11.8 (10.3–13)	0.06	12.3 (11.2–13.7)	11.9 (10.3-13.3)	0.024
Previous aortic valve replacement—no. (%)	27 (3)	23 (3.2)	4 (2.1)	0.404	3 (2.3%)	4 (3.1%)	0.709
Coronary heart disease (CHD)—no. (%)	609 (67.8)	420 (59.5)	189 (100)	<0.001	99 (76.2%)	127 (97.7%)	<0.001
Previous CABG—no. (%)	83 (9.2)	71 (10)	12 (6.3)	0.11	11 (8.5%)	11 (8.5%)	1.000
Dual-antiplatelet therapy—no. (%)	157 (17.5)	48 (6.8)	109 (57.1)	<0.001	48 (36.9%)	48 (36.9%)	1.000
NOAC or VKA—no. (%)	357 (39.8)	269 (38.2)	70 (36.6)	0.693	52 (40.0%)	42 (32.3%)	0.197
Indication for NOAC or VKA				<0.001			
Atrial fibrillation—no. (%)	140 (81.3)	88 (24.8)	52 (45.5)				
Other—no. (%)	307 (118.7)	267 (75.2)	40 (43.5)				
Concomitant PCI—no. (%)			60 (31.4)				

**Table 2:** Procedural Characteristics.

	Protamine Full Dose (N = 706)	Protamine Half Dose (N = 191)	*p*-Value
UFH (mean ± SD)—IU	9302 ± 2976	9024 ± 2471	0.88
Vascular closure device			0.574
Prostar XL—no. (%)	567 (80.3)	157 (82.2)	
MANTA—no. (%)	113 (16)	27 (14.1)	
Perclose ProGlide—no. (%)	5 (0.7)	1 (0.5)	
Stent implantation—no. (%)	52 (7.4)	16 (8.4)	0.629
Additional access site			0.502
A. femoralis—no. (%)	556 (78.8)	159 (83.2)	
A. radialis—no. (%)	134 (19)	29 (15.2)	
Transcatheter heart valve			0.21
CoreValve Evolut R/Pro—no. (%)	619 (88)	172 (91)	
Edwards SAPIEN 3—no. (%)	46 (6.6)	8 (42)	
Acurate NEO—no. (%)	31 (5.2)	9 (4.8)	
PCI access site			
A. femoralis—no. (%)		124 (78)	
A. radialis—no. (%)		35 (22)	

**Table 3:** Primary and Secondary Outcomes Before Propensity Matching.

	Overall (N = 897)	Protamine Full (N = 706)	Protamine Half (N = 191)	*p*-Value
Primary				
Combined endpoint	79 (8.8%)	50 (7.1%)	29 (15.2%)	<0.001
In-hospital mortality	37 (4.1%)	26 (3.7%)	11 (5.8%)	0.200
Life-threatening bleeding	23 (2.5%)	14 (2%)	9 (4.7%)	0.034
Major bleeding	28 (3.1%)	16 (2.3%)	12 (6.3%)	0.005
Major vascular complications	37 (4.1%)	25 (3.5%)	12 (6.3%)	0.091
Secondary				
Stroke after TAVI	15 (1.7%)	11 (1.6%)	4 (2.1%)	0.608
Myocardial Infarction	3 (0.003%)	2 (0.002%)	1 (0.005%)	0.610
Minor bleeding	124 (13.8%)	87 (12.2%)	37 (19.5%)	0.01
Minor vascular complications	204 (22.7%)	157 (22.2%)	48 (25.1%)	0.235
In-hospital stay (days)	17.1 ± 14.1	15.9 ± 12.7	21.7 ± 17.6	<0.001
Blood transfusion volume (units)	0.7 ± 2.0	0.5 ± 1.6	1.1 ± 3.1	<0.001

**Table 4:** Univariable Logistic Regression Before Propensity Matching: Low-Dose Protamine Predicted the Combined Endpoint.

	OR (95%-CI)	*p*-Value
Primary Endpoints		
Combined endpoint	2.35 (1.44–3.82)	<0.001
In-hospital mortality	1.60 (0.77–3.30)	0.204
Life-threatening bleeding	2.44 (1.04–5.74)	0.040
Major bleeding	2.89 (1.34–6.22)	0.007
Major vascular complications	1.83 (0.90–3.71)	0.095
Secondary Endpoints		
Stroke after TAVI	1.35 (0.42–4.29)	0.609
Minor bleeding	1.74 (1.14–2.66)	0.011
Minor vascular complications	1.14 (0.78–1.66)	0.488

**Table 5:** Primary and Secondary Outcomes After Propensity Matching.

	Protamine Full (N = 130)	Protamine Half (N = 130)	*p*-Value
Primary Endpoints			
Combined endpoint	6 (4.6%)	19 (14.6%)	0.006
In-hospital mortality	4 (3.1%)	7 (5.4%)	0.355
Life-threatening bleeding	1 (0.8%)	6 (4.6%)	0.055
Major bleeding	2 (1.5%)	8 (6.2%)	0.053
Major vascular complications	3 (2.3%)	7 (5.4%)	0.197
Secondary Endpoints			
Stroke after TAVI	2 (1.5%)	3 (2.3%)	0.556
Minor bleeding	13 (10%)	27 (20.9%)	0.015
Minor vascular complications	32 (24.6%)	28 (21.5%)	0.652
In-hospital stay	15.3 ± 10.2	20.3 ± 17.4	<0.001
Blood transfusion volume (units)	0.3 ± 0.8	1.2 ± 3.2	0.003

**Table 6:** Univariable Logistic Regression After Propensity Matching: Low-Dose Protamine Predicted the Combined Endpoint.

	OR (95%-CI)	*p*-Value
Primary Endpoints		
Combined endpoint	3.54 (1.36–9.17)	0.009
In-hospital mortality	1.79 (0.51–6.28)	0.361
Life-threatening bleeding	6.24 (0.74–52.59)	0.092
Major bleeding	4.20 (0.87–20.16)	0.073
Major vascular complications	2.41 (0.61–9.53)	0.210
Secondary Endpoints		
Stroke after TAVI	1.51 (0.25–9.20)	0.654
Minor bleeding	2.33 (1.17–4.86)	0.017
Minor vascular complications	0.84 (0.47–1.50)	0.556

## Data Availability

Data are available upon reasonable request to the corresponding author.

## Supplementary Materials

The following supporting information can be downloaded at: https://www.mdpi.com/article/10.3390/jcm12134243/s1, Table S1. Univariable Analysis Before and After Matching, Table S2. Multivariable Analysis of the Combined Endpoint, Table S3. Multivariable Analysis of the Combined Endpoint in Matched Models.

## Abbreviations

TAVI	Transcatheter Aortic Valve Implantation
ACT	Activated Clotting Time
STS	Society of Thoracic Surgeons
PCI	Percutaneous Coronary Intervention
VARC	Valve Academic Research Consortium
DAPT	Dual Antiplatelet Therapy
CKD	Chronic Kidney Disease
BMI	Body Mass Index
GARY	German Aortic Valve Registry
IU	International Units
