# Supplementary material for: Efficacy and Safety of Low-Dose Protamine in Reducing Bleeding Complications during TAVI: A Propensity-Matched Comparison
Source: J Clin Med. 2023 Jun 24;12(13):4243. doi: 10.3390/jcm12134243 (PMC10342582; doi:10.3390/jcm12134243)
Supplement: Supplementary file 1 [file jcm-12-04243-s001.zip › jcm-2404769-supplementary.pdf]

Journal of
Clinical Medicine

MDPI

Supplementary Tables

Table S1. Univariable Analysis Before and After Matching.

	OR (95%-CI)	p	OR (95%-CI)	p
Age (per 10yrs)	0.96 (0.69 - 1.35)	0.830	1.60 (0.78 - 3.23)	0.193
Sex (male)	0.68 (0.43 - 1.09)	0.684	0.49 (0.21 - 1.12)	0.091
EuroScore (per 10%)	1.37 (1.13 - 1.65)	0.001	1.22 (0.85 - 1.76)	0.274
COPD (presence)	0.61 (0.35 - 1.04)	0.067	0.35 (0.12 - 1.07)	0.066
DM (presence)	1.11 (0.69 - 1.79)	0.655	0.58 (0.23 - 1.45)	0.246
Hypertension (presence)	0.82 (0.47 - 1.41)	0.467	0.95 (0.36 - 2.50)	0.917
PAD (presence)	1.68 (0.96 - 2.94)	0.069	1.73 (0.71 - 4.24)	0.230
Cerebral artery disease (presence)	1.13 (0.69 - 1.85)	0.626	0.96 (0.41 - 2.26)	0.920
Smoker (presence)	1.16 (0.58 - 2.33)	0.681	0.90 (0.25 - 3.18)	0.867
Prev. Stroke (presence)	1.82 (0.87 - 3.81)	0.111	1.20 (0.32 - 4.51)	0.782
GFR (per 10 ml/min/1.73 m²)	0.88 (0.78 - 0.99)	0.039	1.02 (0.83 - 1.25)	0.873
LDL (per 10 mg/dl)	0.93 (0.87 - 1.00)	0.069	0.95 (0.83 - 1.09)	0.499
Hb (per g/dl)	0.69 (0.59 - 0.79)	<0.001	0.71 (0.56 - 0.89)	0.004
Previous AV replacement (presence)	1.84 (0.62 - 5.45)	0.273	1.58 (0.18 - 13.71)	0.676
CHD (presence)	1.98 (1.12 - 3.50)	0.018	3.92 (0.51 - 29.97)	0.188
Previous CABG (presence)	0.95 (0.42 - 2.14)	0.900	0.42 (0.05 - 3.30)	0.425
DAPT (presence)	1.68 (0.98 - 2.89)	0.058	1.15 (0.50 - 2.68)	0.738
NOAC or VKA (presence)	0.89 (0.55 - 1.44)	0.641	0,53 (0.20 - 1.37)	0.189
**Low-Dose Protamin (presence)**	**2.35 (1.44 - 3.83)**	**<0.001**	**3.54 (1.36 - 9.17)**	**0.009**

Table S2. Multivariable Analysis of the Combined Endpoint.

EuroScore (per 10%)	1.27 (1.03 - 1.55)	0.022
Hb (per g/dl)	0.71 (0.62 - 0.83)	<0.001
**Low-Dose Protamin (presence)**	**2.07 (1.23 - 3.47)**	**0.006**

In the whole study population, variables successively excluded from the final multivariable regression model bee means of backwards selection were : GFR (p=0.913, 1st round), PAD (p=0.562, 2nd round), LDL (p=0.454, 3rd round), DAPT (p=0.499, 4th round), CAD (p=0.172, 5th round) and COPD (p=0.086, 6th round).

Table S3. Multivariable Analysis of the Combined Endpoint in Matched Models :.

Hb (per g/dl)	0.73 (0.58 - 0.93)	0.010
**Low-Dose Protamin (presence)**	**3.07 (1.17 - 8.08)**	**0.023**

The same analysis as previously described was performed only on the matched population, variables successively eliminated from the regression model were : sex (p=0.229, 1st round) and COPD (p=0.054, 2nd round).
